# Educational technology for multidisciplinary training for managing waiting lists for elective patients

**DOI:** 10.1590/0034-7167-2023-0299

**Published:** 2024-07-29

**Authors:** Rosa Ladi Lisbôa, Kaihara de Freitas Furtado, Vitória Silva da Rosa, Caroline Schacker Evangelista, Adriana Aparecida Paz

**Affiliations:** I Hospital Nossa Senhora da Conceição. Porto Alegre, Rio Grande do Sul, Brazil; II Universidade Federal de Ciências da Saúde de Porto Alegre. Porto Alegre, Rio Grande do Sul, Brazil

**Keywords:** Health Management, Waiting Lists, Educational Technology, eHealth Strategies, Nursing, Gestión en Salud, Listas de Espera, Tecnología Educacional, Estrategias de eSalud, Enfermería

## Abstract

**Objectives:**

to construct and assess an educational technology for managing patient waiting lists for multidisciplinary training.

**Methods:**

study supported by Instructional Design - ADDIE model, whose stages of construction of educational technology were developed in the form of a multi-professional training course. Its respective content assessment was carried out by a committee of experts from 2021 to 2022. The analysis occurred based on the proportion of content adequacy with 95% Confidence Interval.

**Results:**

seventeen products were created as educational technology learning objects: five storyboards; four videos; three comic books; two pedagogical action plans; a mind map; and a YouTube^®^ playlist. Nine experts assessed content adequacy, which reached 0.89.

**Conclusions:**

this educational technology contributes to the performance of professionals who manage waiting lists by reducing inequalities, alleviating differences, in addition to promoting equity in care and good health for patients in the Brazilian Health System.

## RESUMEN

**Objetivos:**

construir y evaluar una tecnología educativa para la gestión de listas de espera de pacientes para formación multidisciplinaria.

**Métodos:**

estudio apoyado en Diseño Instruccional - modelo ADDIE, cuyas etapas de construcción de tecnología educativa se desarrollaron en forma de curso de formación multiprofesional. Su respectiva evaluación de contenidos fue realizada por un comité de expertos del 2021 al 2022. El análisis se realizó utilizando la proporción de adecuación del contenido con un Intervalo de Confianza del 95%.

**Resultados:**

se crearon 17 productos como objetos de aprendizaje de tecnología educativa: cinco *storyboards*; cuatro vídeos; tres cómics; dos planes de acción pedagógicos; un mapa mental; y una lista de reproducción de *YouTube* ^®^. Nueve expertos evaluaron la adecuación de los contenidos, que alcanzó un 0,89.

**Conclusiones:**

esta tecnología educativa contribuye al desempeño de los profesionales que gestionan las listas de espera, reduciendo las desigualdades, aliviando las diferencias, además de promover la equidad en la atención y la buena salud de los pacientes del Sistema de Salud Brasileño.

## INTRODUCTION

The Brazilian Health System (SUS - *Sistema Único de Saúde*), created in 1990 based on the Federal Constitution of 1988, has been continually seeking its consolidation through policies, projects and actions, in order to qualify and increasingly achieve its principles, such as such as accessibility, completeness, equity, efficiency, among others^(1)^. It is possible to state that, since its implementation, the Brazilian public health system has been improved over the decades.

One of the most noticeable problems in society, and which is the target of correct criticism from the population, is the long waiting time for elective (scheduled) surgeries^(2)^. In Brazil, despite the subject being an important phenomenon for the health system, the national literary arsenal found on the subject is scarce^(2-3)^.

Waiting times for elective treatments, including elective surgery, are a source of public concern and therefore a concern for policymakers^(4)^. The average waiting time in several countries is at least three months and can reach years, which generates not only dissatisfaction among patients and the population in general, but also a worsening/deterioration of health conditions, prolongation of suffering, loss of utility/function and even progression to patient death^(5-6)^. Furthermore, there are economic risks, as patients with more serious clinical conditions tend to remain hospitalized for longer and, as a result, require more resources. The fact that these patients are more exposed to, for instance, hospital infections, generates a vicious cycle of increased length of stay and other damages^(4)^.

Among existing weaknesses in health systems, the management of waiting lists for elective surgeries and the lack of professional training in healthcare services stand out. In this regard, it is important to provide training and continuing education of human resources in health, including being constitutional to Brazilian society, and reaffirmed as one of the purposes of Law 8.080/90, which provides for the health system organization^(7)^. This topic is in line with the Sustainable Development Goals (SDGs) of the 2030 Agenda of the United Nations (UN) and the Organization for Economic Cooperation and Development (OECD), which contributes to reducing inequalities; good health and well-being; and quality education^(8)^.

The Internal Regulation Center (IRC) is a sector that seeks to manage the service capacity of health system users in the hospital institution. The experience of one of the authors as a regulatory nurse at a large, general public hospital, located in southern Brazil, enabled reflecting on the work process. The action takes place alongside the sector’s coordination, with a series of practical measures for managing the institution’s lists as: meetings with teams and leaders of medical specialties; joint efforts to carry out pre-operative exams and consultations; monitoring; changes to processes and the information system; and adequacy of use of installed capacity.

This study is based on a Professional Master’s Dissertation that created an educational technology constructed and assessed with technical-scientific rigor. This technology fits into the form of a training course, with a view to qualifying healthcare professionals and administrative technicians who work in regulating services on waiting lists for elective surgical and clinical patients.

## OBJECTIVES

To construct and assess an educational technology for multidisciplinary training for managing patient waiting lists.

## METHODS

### Ethical aspects

The study was approved by the Research Ethics Committee and met the guidelines and regulatory standards for research with human beings. The Informed Consent Form was obtained from all participants who registered acceptance in the Informed Consent Registry (ICR). This document informs on the guarantee of anonymity, confidentiality, privacy and secrecy.

### Study design, period and location

This is a methodological study of technological production, developed in two stages and based on the Instructional Design of the ADDIE model (acronym for Analysis, Design, Development, Implementation and Evaluation)^(9)^. The model has two phases, namely conception and execution. In the conception phase (Analysis, Design and Development), the first stage comprised educational technology construction in the form of a multi-professional training course. The second stage refers to content adequacy assessment by a committee of experts.

The product was developed in a shared (remote) environment aligned with a Graduate Program in Nursing - Professional Master’s Degree at a public university in southern Brazil. The executing team was made up of three nurses (a doctoral holder, a master’s degree holder and a master’s student) and two nursing students (scientific initiation and teaching initiation scholarship holders). The stages took place from March 2021 to October 2022.

### Methodological procedures

The first stage consisted of constructing educational technology products. To begin construction, it is necessary to analyze the demand and need for educational technology, which occurs from collecting information from scientific literature review, the team’s prior knowledge and experience in healthcare services and care and management indicators, such as number of surgeries, elective hospitalizations, monitoring of waiting lists, among others. All of this information provides an opportunity to improve the teaching-learning process for a specific target audience^(9-11)^.

Based on demand analysis, pedagogical design was prepared, which consists of establishing strategies and tools that will be used as well as projection of professionals, costs and schedule for educational technology construction^(9,11)^. A Pedagogical Action Plan (PAP) was structured for each module of the course.

In addition to conducting the theoretical constructs, the PAP guided actions for developing content in accordance with educational objectives. It was then edited into a text document with the following items: course title; total course load; menu; module title; module workload; objective; skills covered in the module; authorship; learning objective; contents; activities/tasks; resources; references; how to cite the material; and update date.

Based on the PAP discussed and approved by all members of the executing team, the course’s visual identity was defined with regard to logography, typography, chromography, pictography and iconography. Subsequently, storyboard construction began, organizing the content and establishing the degree of interactivity with participants, including the different resources used and described in PAP^(9,11-12)^.

The storyboards were structured in the Microsoft Power Point^®^ visual presentation editor in slide show format (*.ppsx), including images and vectors from public photo banks, licensed and royalty-free images and illustrations, such as such as Canva^®^, Flaticon^®^ and Freepik^®^. At the end of the storyboards, references to the images used are described.

The presence of videos in storyboards aimed to make content approach more interactive and dynamic. The educational resources were created using Canva^®^ and Animaker^®^ in the Moving Picture Experts Group (MPEG) 4 Part 14 (*.MP4) format. The videos made available on a research group’s YouTube^®^ channel, under a Creative Commons Attribution 4.0 International license, were indicated in the orderly presentation of the storyboards. The audios were recorded in the Moving Picture Experts Group (MPEG)1/2 Audio Layer 3 (*.MP3) format for the characters created in the Zepeto^®^ and Animaker^®^ applications with the aim of establishing dialogic interaction throughout the course.

The second stage consisted of content adequacy assessment by experts. The process of assessing course content adequacy occurs in the design phase. Between six and 20 experts were stipulated, as recommended in the literature on content assessment processes^(13-14)^. The final sample comprised a committee of experts composed of nine professionals.

The experts could be from any health or education institution in Brazil, as long as they met the inclusion criteria: being a health or administration professional; having specialization in of health management (*lato sensu*) or master’s or doctoral degree (*stricto sensu*) in education, administration, nursing or health sciences; having experience of at least one year working in waiting list management; having publications and/or research in health management; and/or having experience as a participant, tutor, lecturer or content writer in a distance education (DE) course.

To assess PAP and storyboards, the Educational Content Validation Instrument in Health (IVCES - *Instrumento de Validação de Conteúdo Educativo em Saúde*) was used. The instrument consists of 18 questions, in Likert format, organized into three domains: a) objectives; b) structure/presentation; and c) relevance^(15)^. To qualitatively assess the content, a descriptive question was added so that it was possible to record criticisms and/or recommendations. Seven questions were included in the instrument, which was edited in Google Forms^®^.

Expert selection was based on the *Curriculum Lattes* Platform, which used Fehring criteria (minimum score of five) to recruit potential experts to constitute the committee^(16-17)^. These criteria were adapted to include professionals who had experience in managing waiting lists. An invitation to participate was sent by email to the 45 selected experts. The email address for accessing the Google Forms^®^ form was made available in the invitation as well as the ICR, the PAP and the storyboards.

Answers were returned every five days, considering that data collection was electronic. The invited experts who did not answer within the first deadline (ten days) received three new messages reinforcing the invitation to participate (except those who notified that they were not interested in participating in the study).

Data collection took place from August to September 2022. Nine experts participated who electronically accepted the ICR and completely filled out the data collection instrument. Answers were stored in a Google Sheets^®^ spreadsheet, which was transferred to Microsoft Excel^®^ spreadsheet format. Each expert received a unique numeric code to compose the database. In this database, no data was observed that would allow identifying experts, which is a measure to protect participant confidentiality and anonymity.

### Analysis of results, and statistics

When processing the database, answers from nominal to numerical expression for each variable in coding were considered. Subsequently, the data were imported into the Statistical Package for the Social Sciences^®^ to carry out statistical analysis. The calculation of each item was carried out using the proportion of content adequacy with a 95% Confidence Interval, based on the evaluators’ answers. Therefore, the assessment considered a minimum acceptable value of 0.80 for the overall and general domains of the course modules^(14)^. Descriptive data from criticisms and/or recommendations for improvement were organized according to each storyboard and PAP. Each expert was named by the letter “E”, plus the number that appears in the database order (e.g., “E1”, “E2”, ... “E9”). To characterize the experts, descriptive statistics were used. The results were presented in tables.

## RESULTS

The results are presented considering, respectively, the two stages of educational technology development.

### Educational technology product construction

**Figure 1:**
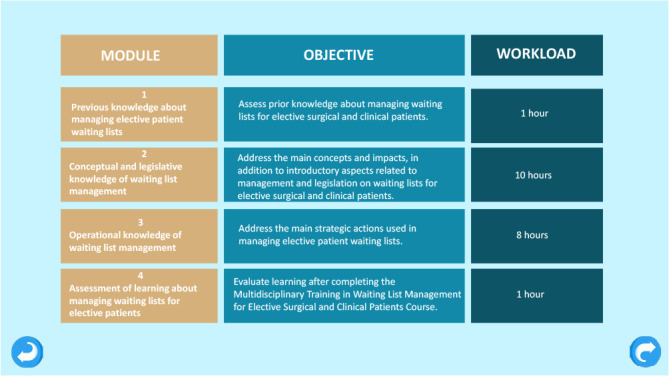
Matrix of multidisciplinary training course in managing patient waiting lists, Porto Alegre, Rio Grande do Sul, Brazil, 2022

The pedagogical design of the educational technology “Multidisciplinary Training in Waiting List Management for Elective Surgical and Clinical Patients Course” was based on the online and self-instructional modality, with a workload of 20 hours. It consisted of two PAP and four storyboards of the course. Figure 1 presents the course matrix containing the modules, objectives and workload.

The preparation of two PAP that support the development of the contents of modules 2 (http://bit.ly/PAPLista2) and 3 (http://bit.ly/PAPLista3) was based on scientific research in the area of list management expectations and experience of executing team members in the topic and educational objectives of the course. The skills to be covered, learning objectives, content, activities and resources were widely discussed and approved by the executing team. In PAP, the various resources that were used to make this teaching-learning process meaningful, dynamic and interactive are described, such as videos, flowcharts, mind maps, comic books, characters, scientific literature for mandatory reading and/or complementary.

The educational technology visual identity and learning resource map were defined and structured on Canva^®^. For each course module, a file was generated, called a storyboard. In total, five storyboards were generated, in which content and activities were developed as well as video, audio, characters, vectors, images and illustrations extracted from public databases. The storyboards were stored on Google Drive^®^, along with the email address for viewing and downloading, but without permission to edit and/or comment.

According to PAP, eight educational resources were planned in different formats to make up the storyboards: four videos; three comic books; a mind map; a playlist on YouTube^®^. Based on the script, four videos were created, one on Animaker^®^ and the others on Canva^®^. The comic books and mind map were also made on Canva^®^. The videos and animated comic books were made available on the research group’s YouTube^®^ channel in a playlist for use in the course or independent access (https://bit.ly/ListaEsperaYouTube), with the playlist being a dynamic and wide-reaching tool, as several people can access it from different places. In addition to the creation of the aforementioned products, the creation and presence of four characters who accompany and present the content to participants stand out. They establish dialogical interaction, which makes the self-instructional course more dynamic and interactive.

In modules 2 and 3, training activities were developed, creating four questions related to theoretical content that were presented at the end of the modules. Modules 1 and 4 are similar in structure. The former contains a diagnostic activity (before content) and the latter has an evaluative activity (after content). These modules do not have PAP for storyboards as they do not include content insertion.

Each of these modules takes one hour to solve three clinical cases that contain seven simple choice questions in four alternatives. Participants can, through clicks, choose the questions to be asked according to their interest. We tried to use different strategies to compose the questions, with a view to diversifying their demands and complexity. The difference between these two modules is in the ordering of clinical cases, fictitious names, geographic location and ordering of correct answers to questions as well as feedback at the end of each question (which did not occur in diagnostic activity). For the purposes of certification of participants in the course, the percentage of correct answers must be equal to or greater than 70% in module 4 assessment activity, in accordance with institutional regulations. Chart 1 presents storyboards of the modules that make up the course as educational technology.

**Chart 1:** Storyboards of multidisciplinary training course modules on managing patient waiting lists, Porto Alegre, Rio Grande do Sul, Brazil, 2022

Module storyboard	Address
1 - Previous knowledge about managing waiting lists for elective patients	http://bit.ly/ListaEspera1
2 - Conceptual and legislative knowledge of waiting list management	http://bit.ly/ListaEspera2
3 - Operational knowledge of waiting list management	http://bit.ly/ListaEspera3
4 - Assessment of learning about managing waiting lists for elective patients	http://bit.ly/ListaEspera4
5 - Course presentation	http://bit.ly/ListaEspera0

These storyboards contain the other learning objects in videos, comic books and mind maps mentioned above. In Chart 1, the “Course presentation” was included, being the fifth storyboard that will be used to customize the virtual learning environment (VLE).

### Content adequacy assessment by experts

The next stage involved the process of assessing content adequacy related to the two PAP and four storyboards of the course modules. The committee of expert was made up of nine participants, who assessed the items according to IVCES.

Of the participants, eight (88.8%) were women. The mean age was 42±6.3 years, and the degrees observed among experts were (6; 66.6%) doctoral and (3; 33.3%) master’s. All experts reported working in a public institution, with six (66.6%) working in higher education and three (33.3%) in healthcare services.

Length of professional experience ranged from two to nine years, with a median of 4.5 (2.7-7.5) years. Among the participants, four reported carrying out concomitant duties in their profession. It was observed that seven (77.7%) work as health managers; five (55.5%) work with hospital care; and one (11.1%) works with health education.

Modules 2 and 3 containing PAP and storyboards were assessed by experts independently, whereas, in modules 1 and 4, storyboard assessment, whose content and complexity were equal, was joint. Table 1 presents the results of experts’ assessment of the content of the four course modules.

**Table 1:** Content assessment of course modules (N=9), Porto Alegre, Rio Grande do Sul, Brazil, 2022

Variables	Modules 1 and 4	Module 2	Module 3
	Proportion (95%CI)	Proportion (95%CI)	Proportion (95%CI)
**Overall assessment**	0.88 (0.76-0.99)	0.93 (0.83 - 1.00)	0.87 (0.73 - 1.00)
**Domain 1 - Objectives**	0.80 (0.67-0.93)	0.93 (0.83 - 1.00)	0.87 (0.73 - 1.00)
Includes proposed topic	1.00 (1.00-1.00)	1.00 (1.00 - 1.00)	1.00 (1.00 - 1.00)
Adequate for the teaching-learning process	1.00 (1.00-1.00)	1.00 (1.00 - 1.00)	0.89 (0.59 - 0.99)
Clarifies doubts about the topic covered	0.56 (0.25-0.83)	0.89 (0.59 - 0.99)	0.78 (0.46 - 0.95)
Provides reflection on the topic	1.00(1.00-1.00)	1.00 (1.00 - 1.00)	1.00 (1.00 - 1.00)
Encourages behavior change	0.44 (0.17-0.75)	0.78 (0.35 - 0.90)	0.67 (0.35 - 0.90)
**Domain 2 - Structure/presentation**	0.89 (0.74 - 1.00)	0.93 (0.83 - 1.00)	0.87 (0.76 - 1.00)
Language adequate to the target audience	0.89 (0.59 - 0.99)	0.89 (0.59 - 0.99)	0.78 (0.46 - 0.95)
Appropriate language for educational material	0.89 (0.59 - 0.99)	1.00 (1.00 - 1.00)	0.89 (0.59 - 0.99)
Interactive language, allowing active involvement in the educational process	0.78 (0.46 - 0.95)	0.78 (0.46 - 0.95)	0.78 (0.46 - 0.95)
Correct information	1.00 (1.00 -1.00)	1.00 (1.00 - 1.00)	0.89 (0.59 - 0.99)
Objective information	0.78 (0.46 - 0.95)	0.89 (0.59 - 0.99)	0.89 (0.59 - 0.99)
Clarifying information	0.78 (0.46 - 0.95)	0.89 (0.59 - 0.99)	0.78 (0.46 - 0.95)
Necessary information	0.78 (0.46 - 0.95)	0.89 (0.59 - 0.99)	0.89 (0.59 - 0.99)
Logical sequence of ideas	1.00 (1.00 - 1.00)	1.00 (1.00 - 1.00)	1.00 (1.00 - 1.00)
Current topic	1.00 (1.00 - 1.00)	1.00 (1.00 - 1.00)	1.00 (1.00 - 1.00)
Appropriate text size	1.00 (1.00 - 1.00)	1.00 (1.00 - 1.00)	0.89 (0.59 - 0.99)
**Domain 3 - Relevance**	0.96 (0.88 - 1.00)	0.93 (0.83 - 1.00)	0.85 (0.63 - 1.00)
Encourages learning	0.89 (0.59 - 0.99)	0.89 (0.59 - 0.99)	0.78 (0.46 - 0.95)
Contributes to knowledge in the area	1.00 (1.00 - 1.00)	0.89 (0.59 - 0.99)	1.00 (1.00 - 1.00)
Arouses interest in the topic	1.00 (1.00 - 1.00)	1.00 (1.00 - 1.00)	0.78 (0.46 - 0.95)

All modules that make up educational technology and were assessed by experts obtained a content adequacy of 0.89 (0.78 - 1.00). This value indicates the quality of adequacy of the course content that will be offered. Modules 1 and 4 were assessed together and obtained an overall evaluation with the same value as module 3, but both have a lower value than that achieved by module 2, which is the most consistent in content and has a greater workload. In relation to the objective and structure/presentation domains, module 2 stood out with the highest value compared to the other modules. In the relevance domain, modules 1 and 4 obtained the best assessment.

Participants described suggestions or recommendations to improve the quality of presentation of thematic content. All of them were welcomed, discussed, identified and adjusted in PAP and storyboards. Some of them are illustrated, such as the one related to didactics, [...] *having some brief questions relating the topic to the practice and daily life of services throughout the module with the possibility of answers and feedback so that each student can go reflecting on what you already know about the topic*. (E7) and [...] *enlightening, interactive and creative, motivating participants to the learning process. All the features (videos, streams and comic books) look fantastic!* (E9).

## DISCUSSION

Waiting list management must involve the participation of several actors. Multidisciplinary integration results in improved patient care^(4,18)^. Furthermore, managing multidisciplinary waiting lists increases the guarantee of fairness and transparency in the elective patient care process. The existence of the list constitutes an overall problem for public healthcare services indicated by OECD^(5-6,8)^, but not for private services, as they raise financial resources for each hospitalization and/or procedure performed.

Along with the lack of specific legal guidelines, there is the problem of scarcity of literary production on the topic^(2-3)^. Thus, it is reflected that, although waiting lists are not a recent problem at a national or even international level in Brazil, negotiations on this issue are still incipient. This further reinforces the need for educational actions for professionals who work in list management.

To truly make sense, professional education must be capable of promoting effective and significant transformations in care practices and in the organization of work processes in healthcare services. Professional education in hospital institutions must be developed in a permanent, active and consolidated manner, contributing to qualifying patient care^(19-20)^. Therefore, the need for educational actions is highlighted with the aim of encouraging, monitoring and strengthening the professional qualifications of workers working in the health sector, aiming at transforming healthcare practices based on the SUS fundamental principles.

Educational technology was structured for VLE, which sought to involve collaborative learning principles. It is hoped that participants become involved in their learning, based on the real situations that were created and that the course facilitates articulation of content with the context of participants’ work^(21-22)^.

Planning was outlined in two PAP that made it possible to define the objectives to be achieved by course participants, the content developed and the learning objects used in each module (including the strategies used to assess users’ progress). Updated scientific literature and target audience experiences are considered as subsidies for defining content in PAP^(9,11)^.

It is noteworthy that, in self-instructional and online courses, the elements specific to the distance learning modality must be considered, even during planning, such as the selection of technologies and visual identity that will be used and that must be included in the description in resources in PAP^(23-24)^. For this reason, since the topic of waiting list management is a complex subject, efforts were made to develop learning objects that enable more participant dynamics and engagement in the course.

Thus, scientific literature mentions that storyboards are important in educational technology construction, as they organize and structure content and activities in a logical sequence in the development stage^(10)^. All course storyboards were assessed by a committee of experts.

The educational technology proposal covered Analysis, Design and Development stages, according to the ADDIE model, which generated 17 products. These are considered to be learning objects that support the multidisciplinary course’s pedagogical proposal.

It is worth emphasizing that, if in the learning process, content is constructed through words and images that sharpen hearing and vision, it is possible to make learning more meaningful than if there were only a focus on reading on screen^(25)^. It is recommended that educational resource production in format of videos designed to form courses does not exceed ten minutes in length^(26)^. In consideration of this instruction, none of the four videos and two animated comic books created for the course exceeded this duration. All videos are grouped in a playlist called “*Complexo Regulador de Saúde*”, available on the research group’s YouTube^®^ channel.

The assessment process of a committee of experts provides careful and careful support for planned educational actions^(10)^. It is noteworthy that PAP and course storyboards prepared reached the value of content adequacy recommended by the literature^(14)^.

A study that assessed digital educational resources for health and safety at work in Primary Health Care and that also used IVCES found values between 0.88 and 0.96, which demonstrates the quality of the course^(27)^. The assessment of the development of a course aimed at using mental health diagnoses and interventions in clinical-surgical hospitalization units obtained values from 0.84 to 1.00^(28)^. Another study concluded that experts who assessed an online course on neonatal pain highlighted the need to construct content and make educational products available independently, allowing users to decide the itinerary of their study^(29)^.

However, the items included in the IVCES domains are important, as they provide a basis for various aspects relevant to educational content, ensuring the offering of a product adequate for the target audience^(15)^. Thus, experts’ suggestions, criticisms and praise contributed to the quality of the proposed course.

All learning objects produced by this study can be (re)used according to the installed need, in addition to professionals having autonomy over the sequence of content they wish to learn in the course. Furthermore, using technologies in education generates greater cognitive gains for participants compared to conventional teaching^(30-31)^.

The study demonstrated relevance when offered to professionals who work in waiting list management, enabling them to understand the concepts, reflect on the impacts and improve operational knowledge related to waiting list management of elective surgical and clinical patients.

### Study limitations

As a challenge experienced in this study, we can mention the ADDIE model design stage, in which many hours of work were required to deliver the presented product. This required an organization of the executing team that worked remotely, given the scope of the pandemic and compliance with safe distancing standards during the construction period of the educational technology.

The lack of time to complete all phases of the ADDIE model, which includes the uncompleted execution phase, was another limiting factor. Two other factors contributed to intensifying the level of difficulty of the course proposal: the fact that the issue addressed still presents an incipient scientific literature; and the practice of list management does not have a common organizational process in all healthcare services.

### Contributions to nursing and health

Based on the aforementioned limiting and hindering factors, it is important to offer the course to healthcare professionals, as using this educational technology in VLE makes it possible to clarify, update and inspire new practices in managing waiting lists. Thus, this educational technology contributes to the practice of different professional categories, especially regulatory nurses, qualifying skills in the nationwide management of elective patient waiting lists.

The practices of regulating and managing waiting lists for nurses working in IRC are recent. however, it is necessary to discuss this topic during the academic training period, when it comes to nursing and health management. It is understood that nurses have the managerial and leadership competence to mediate the list regulation process, as it strengthens the analysis of indicators and the formulation of strategies in the institution^(4)^. Furthermore, it is noteworthy that educational technologies enable qualifying nurses and other professionals so that these professionals can participate and contribute in sectors in which work is multidisciplinary.

## CONCLUSIONS

Educational technology construction and assessment for multidisciplinary training achieved the quality of the content presented, using different strategies to create learning objects that were interrelated in the course’s pedagogical composition. Offering the course in its self-instructional modality favors the expansion of knowledge, as it allows professionals who work in managing waiting lists autonomy in the teaching-learning process. Furthermore, it contributes to developing and improving the work process so that bed regulation is carried out in a more qualified and efficient manner.

The participation of experts in course module assessment was essential to qualify the educational technology as well as scientifically guaranteeing, through a scale, content adequacy associated with learning objects. It is understood that, for improvement and updating to occur, educational technologies based on technical-scientific rigor must be encouraged and available.

It is considered that this educational technology should be aimed at students, residents and professionals, especially those who work in public healthcare institutions. Such institutions may be commonly marked by disparity in management and conditions between regions of Brazil. Hence, the multidisciplinary training course as an educational technology can collaborate with SDGs to reduce inequalities, alleviate differences, in addition to promoting equity in care and good health for patients.
