# Supplementary material for: Educational technology for multidisciplinary training for managing waiting lists for elective patients
Source: Rev Bras Enferm. 2024 Jul 29;77(3):e20230299. doi: 10.1590/0034-7167-2023-0299 (PMC11290741; doi:10.1590/0034-7167-2023-0299)
Supplement: Translation

# Translation: Tecnologia educativa para a formação multiprofissional no gerenciamento de listas de espera de pacientes eletivos

**DOI:** 10.1590/0034-7167-2023-0299pt

**Authors:** Rosa Ladi Lisbôa, Kaihara de Freitas Furtado, Vitória Silva da Rosa, Caroline Schacker Evangelista, Adriana Aparecida Paz

**Affiliations:** I Hospital Nossa Senhora da Conceição. Porto Alegre, Rio Grande do Sul, Brasil; II Universidade Federal de Ciências da Saúde de Porto Alegre. Porto Alegre, Rio Grande do Sul, Brasil

**Keywords:** Gestão em Saúde, Listas de Espera, Tecnologia Educacional, Estratégias de eSaúde, Enfermagem

## Abstract

**Objetivos:**

construir e avaliar uma tecnologia educativa de gerenciamento de listas de espera de pacientes para a formação multiprofissional.

**Métodos:**

estudo sustentado pelo *Design* Instrucional - modelo ADDIE, cujas etapas de construção da tecnologia educativa foram desenvolvidas na modalidade de curso de formação multiprofissional. Sua respectiva avaliação de conteúdo foi realizada por um comitê de especialistas de 2021 a 2022. A análise ocorreu pela proporção de adequação de conteúdo com Intervalo de Confiança de 95%.

**Resultados:**

elaboraram-se 17 produtos como objetos de aprendizagem da tecnologia educativa: cinco *storyboards*; quatro vídeos; três histórias em quadrinhos; dois planos de ação pedagógica; um mapa mental; e uma *playlist* do *YouTube* ^®^. Nove especialistas avaliaram adequação de conteúdo, que atingiu 0,89.

**Conclusões:**

essa tecnologia educativa contribui na atuação dos profissionais que gerenciam listas de espera ao reduzir desigualdades, amenizar diferenças, além de promover equidade no atendimento e boa saúde ao paciente no Sistema Único de Saúde.

## INTRODUÇÃO

O Sistema Único de Saúde (SUS), criado em 1990 a partir da Constituição Federal de 1988, vem buscando continuamente a sua consolidação por meio de políticas, projetos e ações, de forma a qualificar-se e alcançar cada vez mais seus princípios, tais como acessibilidade, integralidade, equidade, eficiência, entre outros^(1)^. É possível afirmar que, desde sua implantação, o sistema de saúde público brasileiro vem sendo aprimorado ao longo das décadas.

Um dos problemas mais perceptíveis à sociedade, e que é alvo de críticas corretas por parte da população, é o longo tempo de espera para a realização de cirurgias eletivas (programadas)^(2)^. No Brasil, apesar de o assunto ser um fenômeno importante para o sistema de saúde, o arsenal literário nacional encontrado sobre o tema é escasso^(2-3)^.

O tempo de espera para tratamentos eletivos, incluindo cirurgia eletiva, é uma fonte de preocupação pública e, portanto, temário para os formuladores de políticas^(4)^. O tempo médio de espera em vários países é de, pelo menos, três meses, podendo chegar a anos, o que gera não apenas insatisfação dos pacientes e da população em geral, mas uma piora/deterioração das condições de saúde, prolongamento do sofrimento, perda de utilidade/função e, inclusive, evolução para o óbito do paciente^(5-6)^. Ademais, existem os riscos econômicos, pois pacientes com quadros clínicos mais graves tendem a permanecer mais tempo internados e, com isso, precisam de mais recursos. O fato de esses pacientes estarem mais expostos, por exemplo, às infecções hospitalares, gera um ciclo vicioso de aumento de permanência e outros danos^(4)^.

Entre as fragilidades existentes nos sistemas de saúde, destacam-se o gerenciamento de listas de espera para cirurgias eletivas e a insuficiência de formação profissional nos serviços de saúde. Nesse sentido, ressalta-se a importância da formação e educação continuada dos recursos humanos em saúde, sendo, inclusive, constitucional à sociedade brasileira e reafirmada como uma das finalidades da Lei n^o^. 8.080/90, que dispõe sobre organização do sistema de saúde^(7)^. Esse temário coaduna-se aos Objetivos de Desenvolvimento Sustentável (ODS) da Agenda 2030 da Organização das Nações Unidas (ONU) e da Organização para a Cooperação e Desenvolvimento Econômico (OECD), que contribui para a redução das desigualdades; boa saúde e bem-estar; e educação de qualidade^(8)^.

O Núcleo Interno de Regulação (NIR) é um setor que busca gerenciar a capacidade de atendimento de usuários do sistema de saúde na instituição hospitalar. A experiência de uma das autoras como enfermeira reguladora de um hospital público, geral e de grande porte, localizado no sul do Brasil, possibilitou reflexões sobre o processo de trabalho. A atuação ocorre junto da coordenação do setor, com uma série de medidas práticas para o gerenciamento das listas da instituição como: reuniões com equipes e lideranças de especialidades médicas; mutirões para a realização de exames e consultas pré-operatórias; monitoramento; alterações de processos e no sistema de informações; e adequação do uso da capacidade instalada.

Este estudo baseia-se na Dissertação de Mestrado Profissional que criou uma tecnologia educativa construída e avaliada com rigor técnico-científico. Essa tecnologia enquadra-se na modalidade de curso de formação, com vistas a qualificar os profissionais de saúde e técnicos administrativos que atuam em serviços de regulação em listas de espera de pacientes cirúrgicos e clínicos eletivos.

## OBJETIVOS

Construir e avaliar uma tecnologia educativa para a formação multiprofissional no gerenciamento de listas de espera de pacientes.

## MÉTODOS

### Aspectos éticos

O estudo foi aprovado por Comitê de Ética em Pesquisa e atendeu às diretrizes e normas regulamentadoras de pesquisas com seres humanos. O Termo de Consentimento Livre e Esclarecido foi obtido de todos os participantes que registraram o aceite no Registro de Consentimento Livre e Esclarecido (RCLE). Esse documento informou sobre a garantia do anonimato, confidencialidade, privacidade e sigilo.

### Desenho, período e local do estudo

Estudo metodológico de produção tecnológica, desenvolvido em duas etapas e alicerçado no *Design* Instrucional do modelo ADDIE (acrônimo em inglês parae Análise, Desenho, Desenvolvimento, Implementação e Avaliação)^(9)^. O modelo possui duas fases, sendo elas a de concepção e a de execução. Na fase de concepção (Análise, Desenho e Desenvolvimento), a primeira etapa compreendeu a construção da tecnologia educativa na modalidade de curso de formação multiprofissional. Já a segunda etapa refere-se à avaliação de adequação do conteúdo por um Comitê de Especialistas.

O produto foi desenvolvido em ambiente compartilhado (remoto) alinhado a um Programa de Pós-Graduação em Enfermagem - Mestrado Profissional de uma universidade pública do sul do Brasil. A equipe executora foi composta por três enfermeiros (uma doutora, uma mestra e uma mestranda) e duas estudantes de enfermagem (bolsistas de iniciação científica e iniciação à docência). As etapas ocorreram no período de março de 2021 a outubro de 2022.

### Procedimentos metodológicos

A primeira etapa consistiu na construção dos produtos da tecnologia educativa. Para iniciar a construção, é necessário analisar a demanda e a necessidade de uma tecnologia educacional, o que ocorre a partir da coleta de informações da revisão da literatura científica, do conhecimento prévio e experiência da equipe nos serviços de saúde e de indicadores assistenciais e gerenciais, tais como número de cirurgias, internações eletivas, monitoramento das listas de espera, entre outros. O conjunto dessas informações oportuniza a melhoria no processo de ensino-aprendizagem de um público-alvo específico^(9-11)^.

Com base na análise de demanda, elaborou-se o desenho pedagógico, que consiste no estabelecimento de estratégias e ferramentas que serão utilizadas, bem como de projeção de profissionais, custos e cronograma para a construção da tecnologia educativa^(9,11)^. Estruturou-se o Plano de Ação Pedagógica (PAP) para cada módulo do curso.

Além de conduzir os construtos teóricos, o PAP orientou as ações para o desenvolvimento do conteúdo de acordo com os objetivos educacionais. A seguir, foi editado em um documento de texto com os seguintes itens: título do curso; carga horária total do curso; ementa; título do módulo; carga horária do módulo; objetivo; competências abrangidas no módulo; autoria; objetivo da aprendizagem; conteúdos; atividades/tarefas; recursos; referências; como citar o material; e data de atualização.

A partir dos PAP discutidos e aprovados por todos os membros da equipe executora, definiu-se a identidade visual do curso no que diz respeito à logografia, tipografia, cromografia, pictografia e iconografia. Posteriormente, deu-se início à construção dos *storyboards*, que organizam os conteúdos e estabelecem o grau de interatividade com o participante, incluindo os diferentes recursos utilizados e descritos no PAP^(9,11-12)^.

Os *storyboards* foram estruturados no editor de apresentação visual do *Miscrosoft PowerPoint* ^®^ em formato de apresentação de *slides* (*.ppsx), sendo incluídos imagens e vetores de bancos públicos de fotos, imagens e ilustrações licenciadas e isentas de *royalties*, tais como *Canva* ^®^, *Flaticon* ^®^ e *Freepik* ^®^. No final dos *storyboards*, são descritas as referências das imagens utilizadas.

A presença de vídeos nos *storyboards* visou tornar a abordagem dos conteúdos mais interativa e dinâmica. Os recursos educativos foram elaborados no *Canva* ^®^ e no *Animaker* ^®^ no formato *Moving Picture Experts Group* (MPEG) 4 Part 14 (*.MP4). Os vídeos disponibilizados no canal do *YouTube* ^®^ de um grupo de pesquisa, sob licença *Creative Commons* Atribuição 4.0 Internacional, foram indicados na apresentação ordenada dos *storyboards*. Os áudios foram gravados no formato *Moving Picture Experts Group* (MPEG)1/2 *Audio Layer* 3 (*.MP3) para as personagens criadas nos aplicativos *Zepeto* ^®^ e *Animaker* ^®^ com o objetivo de estabelecer a interação dialógica no transcorrer do curso.

A segunda etapa consistiu na avaliação da adequação de conteúdo por especialistas. O processo de avaliação da adequação de conteúdo do curso ocorreu na fase de concepção. Estipularam-se entre seis e 20 especialistas, conforme recomenda a literatura em processos de avaliação de conteúdo^(13-14)^. A amostra final compreendeu um comitê de especialistas composto por nove profissionais.

Os especialistas poderiam ser de qualquer instituição de saúde ou de educação no Brasil, desde que atendessem aos critérios de inclusão, como ser profissional de saúde ou da área da administração; ter especialização na área da gestão em saúde (*lato sensu*) ou mestrado ou doutorado (*stricto sensu*) nas áreas da educação, administração, enfermagem, ou ciências da saúde; ter experiência de, no mínimo, um ano atuando no gerenciamento de listas de espera; possuir publicações e/ou pesquisa na área da gestão em saúde; e/ou ter experiência como participante, tutor, ministrante ou conteudista em curso em Educação a Distância (EaD).

Para avaliação dos PAP e *storyboards*, utilizou-se o Instrumento de Validação de Conteúdo Educativo em Saúde (IVCES). O instrumento é composto por 18 questões, em formato Likert, organizadas em três domínios: a) objetivos; b) estrutura/apresentação; e c) relevância^(15)^. Para avaliar de forma qualitativa o conteúdo, acrescentou-se uma questão descritiva, de maneira que fosse possível realizar o registro de críticas e/ou recomendações. Sete questões foram incluídas ao instrumento, que foi editado no *Google Forms* ^®^.

A seleção dos especialistas baseou-se na Plataforma do *Curriculum Lattes*, que utilizou os critérios de Fehring (pontuação mínima de cinco) para recrutar os potenciais especialistas para constituir o comitê^(16-17)^. Esses critérios foram adaptados com vistas a contemplar os profissionais que possuíam experiência em gerenciamento de listas de espera. O convite para participação foi encaminhado por correio eletrônico aos 45 especialistas selecionados. O endereço eletrônico para o acesso formulário do *Google Forms* ^®^ foi disponibilizado no convite, assim como o RCLE, os PAP e os *storyboards*.

A cada cinco dias, verificou-se o retorno das respostas, tendo em vista que a coleta de dados foi eletrônica. Os especialistas convidados que não responderem no primeiro prazo concedido (de dez dias) receberam três novas mensagem reforçando o convite para participar (exceto aqueles que notificaram não ter interesse em participar do estudo).

A coleta de dados ocorreu no período de agosto a setembro de 2022. Participaram nove especialistas que aceitaram eletronicamente o RCLE e preencheram completamente o instrumento de coleta de dados. As respostas foram armazenadas em uma planilha do *Google Sheets* ^®^, que foi transferida para o formato de planilha do *Microsoft Excel* ^®^. Cada especialista recebeu um código numérico único para compor o banco de dados. Nesse banco, não foram observados dados que possibilitassem a identificação dos especialistas, sendo essa uma medida de proteção ao sigilo e anonimato dos participantes.

### Análise dos resultados e estatística

No tratamento do banco de dados, foram consideradas as respostas de expressão nominal para numérica de cada variável na codificação. Posteriormente, os dados foram importados para o *Statistical Package for the Social Sciences* ^®^ para se proceder à análise estatística. O cálculo de cada item foi realizado pela proporção de adequação de conteúdo com Intervalo de Confiança de 95%, a partir das respostas dos avaliadores. Portanto, a avaliação considerou como valor aceitável mínimo de 0,80 para os domínios, global e geral dos módulos do curso^(14)^. Os dados descritivos provenientes de críticas e/ou recomendações para melhoria foram organizados de acordo com cada *storyboard* e PAP. Cada especialista foi nominado pela letra “E”, acrescida do número que consta no ordenamento do banco de dados (por exemplo, “E1”, “E2”, ... “E9”). Para a caracterização dos especialistas, utilizou-se estatística descritiva. Os resultados foram apresentados em tabelas.

## RESULTADOS

Os resultados foram apresentados considerando-se, respectivamente, as duas etapas de desenvolvimento da tecnologia educativa.

### Construção dos produtos da tecnologia educativa

O desenho pedagógico da tecnologia educativa “Curso de Formação Multiprofissional em Gerenciamento de Listas de Espera” foi assentado na modalidade *online* e autoinstrucional, com carga horária de 20 horas. Ele foi constituído por dois PAP e quatro *storyboards* do curso. A Figura 1 apresenta a matriz do curso contendo os módulos, objetivos e carga horária.

**Figura 1:** Matriz do curso de formação multiprofissional no gerenciamento de listas de espera de pacientes, Porto Alegre, Rio Grande do Sul, Brasil, 2022

A elaboração de dois PAP, que subsidiam o desenvolvimento dos conteúdos dos módulos 2 (http://bit.ly/PAPLista2) e 3 (http://bit.ly/PAPLista3), foi embasada na pesquisa científica na área da gestão das listas de espera e na experiência dos membros da equipe executora no tema e nos objetivos educacionais do curso. As competências a serem abrangidas, os objetivos de aprendizagem, os conteúdos, as atividades e os recursos foram amplamente discutidos e aprovados pela equipe executora. Nos PAP, estão descritos os diversos recursos que foram utilizados para tornar significativo, dinâmico e interativo esse processo de ensino-aprendizagem, tais como vídeos, fluxogramas, mapa mental, histórias em quadrinhos (HQ), personagens, literatura científica para leitura obrigatória e/ou complementar.

A identidade visual e o mapa de recursos de aprendizagem da tecnologia educativa foram definidos e estruturados na plataforma *Canva* ^®^. Para cada módulo do curso, foi gerado um arquivo denominado *storyboard.* No total, foram gerados cinco *storyboards*, nos quais foram desenvolvidos os conteúdos e as atividades, assim como foram incorporados os recursos de vídeo, áudios, personagens, vetores, imagens e ilustrações extraídas de bancos públicos. Os *storyboards* foram armazenados no *Google Drive* ^®^ juntamente com o endereço eletrônico para visualização e *download*, mas sem a permissão de edição e/ou comentário.

De acordo com os PAP, oito recursos educativos foram planejados em diferentes formatos para compor os *storyboards*: quatro vídeos; três HQ; um mapa mental; uma *playlist* na plataforma do *YouTube* ^®^. A partir da roteirização, estabeleceu-se a criação de quatro vídeos, sendo um no *Animaker* ^®^ e os demais no *Canva* ^®^. As HQ e o mapa mental também foram feitos no *Canva* ^®^. Os vídeos e HQ animadas foram disponibilizados no canal de *YouTube* ^®^ do grupo de pesquisa em uma *playlist* para o uso no curso ou acesso independente (https://bit.ly/ListaEsperaYouTube), sendo a *playlist* uma ferramenta dinâmica e de amplo alcance, pois várias pessoas podem acessá-la de diferentes lugares. Além da criação dos produtos supracitados, destacam-se a criação e a presença de quatro personagens que acompanham e apresentam o conteúdo ao participante. Elas estabelecem a interação dialógica, o que torna o curso autoinstrucional mais dinâmico e interativo.

Nos módulos 2 e 3, foram elaboradas atividades formativas, sendo criadas quatro questões relacionadas ao conteúdo teórico que foram apresentadas ao final dos módulos. Os módulos 1 e 4 são semelhantes em sua estrutura. O primeiro contém uma atividade diagnóstica (antes do conteúdo), e o último possui uma atividade avaliativa (depois do conteúdo). Esses módulos não possuem os PAP para *storyboards*, por não terem a inserção de conteúdo.

Cada um desses módulos possui a carga horária de uma hora para a resolução de três casos clínicos que contêm sete questões de escolha simples em quatro alternativas. O participante pode, por meio de cliques, escolher as questões a serem realizadas de acordo com o seu interesse. Procurou-se utilizar diferentes estratégias para compor as questões, com vistas a diversificar as exigências e complexidade das mesmas. A diferença existente entre esses dois módulos está no ordenamento dos casos clínicos, nos nomes fictícios, na localização geográfica e no ordenamento das respostas corretas das perguntas, bem como no *feedback* ao final de cada questão (que não ocorreu na atividade diagnóstica). Para fins de certificação do participante no curso, é preciso que o percentual de acertos seja igual ou superior a 70% na atividade avaliativa do módulo 4, conforme o regulamento institucional. O Quadro 1 apresenta *storyboards* dos módulos que compõem o curso como tecnologia educativa.

**Quadro 1:** Storyboards dos módulos do curso de formação multiprofissional no gerenciamento de listas de espera de pacientes, Porto Alegre, Rio Grande do Sul, Brasil, 2022

*Storyboard* dos módulos	Endereço
1 - Conhecimentos prévios sobre gerenciamento das listas de espera de pacientes eletivos	http://bit.ly/ListaEspera1
2 - Conhecimentos conceituais e legislativos da gestão de listas de espera	http://bit.ly/ListaEspera2
3 - Conhecimento operacional sobre gestão de listas de espera	http://bit.ly/ListaEspera3
4 - Avaliação da aprendizagem sobre gerenciamento de listas de espera de pacientes eletivos	http://bit.ly/ListaEspera4
5 - Apresentação do curso	http://bit.ly/ListaEspera0

Nesses *storyboards,* estão contidos os demais objetos de aprendizagem em formato de vídeo, HQ e mapa mental citados anteriormente. No Quadro 1, foi incluída a “Apresentação do curso”, sendo o quinto *storyboard* que será utilizado para a customização do ambiente virtual de aprendizagem (AVA).

**Tabela 1:** Avaliação de conteúdo dos módulos do curso (N=9), Porto Alegre, Rio Grande do Sul, Brasil, 2022

Variáveis	Módulos 1 e 4	Módulo 2	Módulo 3
	Proporção (IC95%)	Proporção (IC95%)	Proporção (IC95%)
**Avaliação global**	0,88 (0,76-0,99)	0,93 (0,83 - 1,00)	0,87 (0,73 - 1,00)
**Domínio 1 - Objetivos**	0,80 (0,67-0,93)	0,93 (0,83 - 1,00)	0,87 (0,73 - 1,00)
Contempla tema proposto	1,00 (1,00-1,00)	1,00 (1,00 - 1,00)	1,00 (1,00 - 1,00)
Adequado ao processo de ensino-aprendizagem	1,00 (1,00-1,00)	1,00 (1,00 - 1,00)	0,89 (0,59 - 0,99)
Esclarece dúvidas sobre o tema abordado	0,56 (0,25-0,83)	0,89 (0,59 - 0,99)	0,78 (0,46 - 0,95)
Proporciona reflexão sobre o tema	1,00(1,00-1,00)	1,00 (1,00 - 1,00)	1,00 (1,00 - 1,00)
Incentiva mudança de comportamento	0,44 (0,17-0,75)	0,78 (0,35 - 0,90)	0,67 (0,35 - 0,90)
**Domínio 2 - Estrutura/apresentação**	0,89 (0,74 - 1,00)	0,93 (0,83 - 1,00)	0,87 (0,76 - 1,00)
Linguagem adequada ao público-alvo	0,89 (0,59 - 0,99)	0,89 (0,59 - 0,99)	0,78 (0,46 - 0,95)
Linguagem apropriada ao material educativo	0,89 (0,59 - 0,99)	1,00 (1,00 - 1,00)	0,89 (0,59 - 0,99)
Linguagem interativa, permitindo envolvimento ativo no processo educativo	0,78 (0,46 - 0,95)	0,78 (0,46 - 0,95)	0,78 (0,46 - 0,95)
Informações corretas	1,00 (1,00 -1,00)	1,00 (1,00 - 1,00)	0,89 (0,59 - 0,99)
Informações objetivas	0,78 (0,46 - 0,95)	0,89 (0,59 - 0,99)	0,89 (0,59 - 0,99)
Informações esclarecedoras	0,78 (0,46 - 0,95)	0,89 (0,59 - 0,99)	0,78 (0,46 - 0,95)
Informações necessárias	0,78 (0,46 - 0,95)	0,89 (0,59 - 0,99)	0,89 (0,59 - 0,99)
Sequência lógica das ideias	1,00 (1,00 - 1,00)	1,00 (1,00 - 1,00)	1,00 (1,00 - 1,00)
Tema atual	1,00 (1,00 - 1,00)	1,00 (1,00 - 1,00)	1,00 (1,00 - 1,00)
Tamanho do texto adequado	1,00 (1,00 - 1,00)	1,00 (1,00 - 1,00)	0,89 (0,59 - 0,99)
**Domínio 3 - Relevância**	0,96 (0,88 - 1,00)	0,93 (0,83 - 1,00)	0,85 (0,63 - 1,00)
Estimula o aprendizado	0,89 (0,59 - 0,99)	0,89 (0,59 - 0,99)	0,78 (0,46 - 0,95)
Contribui para o conhecimento na área	1,00 (1,00 - 1,00)	0,89 (0,59 - 0,99)	1,00 (1,00 - 1,00)
Desperta interesse pelo tema	1,00 (1,00 - 1,00)	1,00 (1,00 - 1,00)	0,78 (0,46 - 0,95)

### Avaliação da adequação de conteúdo por especialistas

A etapa seguinte envolveu o processo de avaliação da adequação do conteúdo relacionado aos dois PAP e quatro *storyboards* dos módulos do curso. O comitê de especialistas foi constituído por nove participantes, que avaliaram os itens de acordo com o IVCES.

Dos participantes, oito (88,8%) eram mulheres. A idade média foi de 42±6,3 anos, e a titulação observada entre os especialistas foi de (6; 66,6%) doutor e (3; 33,3%) mestre. Todos os especialistas referiram atuar em uma instituição de natureza pública, sendo que seis (66,6%) laboram na área da educação superior e três (33,3%) em serviços na área da saúde.

O tempo de experiência profissional variou de dois a nove anos, com uma mediana de 4,5 (2,7-7,5) anos. Entre os participantes, quatro assinalaram exercer na sua profissão atribuições concomitantes. Observou-se que sete (77,7%) atuam como gestor na área da saúde; cinco (55,5%) trabalham com assistência hospitalar; e um (11,1%) labora com a educação em saúde.

Os módulos 2 e 3 contendo os PAP e *storyboards* foram avaliados pelos especialistas de forma independente, enquanto que, nos módulos 1 e 4, a avaliação dos *storyboards*, cujo conteúdo e complexidade eram iguais, foi conjunta. A Tabela 1 apresenta os resultados da avaliação dos especialistas sobre o conteúdo dos quatro módulos do curso.

Todos os módulos que compõem a tecnologia educativa e foram avaliados pelos especialistas obtiveram a adequação de conteúdo de 0,89 (0,78 - 1,00). Esse valor indica a qualidade da adequação do conteúdo do curso que será oferecido. Os módulos 1 e 4 foram avaliados conjuntamente e obtiveram a avaliação global com valor igual ao módulo 3, mas ambos possuem o valor inferior ao atingido pelo módulo 2, que é o mais consistente em conteúdo e possui maior carga horária. Em relação aos domínios objetivo e estrutura/apresentação, destacou-se o módulo 2 com o maior valor, em comparação aos demais módulos. No domínio relevância, os módulos 1 e 4 obtiveram a melhor avaliação.

Os participantes descreveram sugestões ou recomendações no sentindo de ampliar a qualidade da apresentação do conteúdo temático. Todas elas foram acolhidas, discutidas, identificadas e ajustadas nos PAP e *storyboards*. Ilustram-se algumas delas, como a que se relaciona à didática, [...] *ter algumas perguntas breves relacionando o tema à prática e ao cotidiano dos serviços ao longo do módulo com possibilidade de resposta e feedback para que cada cursista possa ir refletindo sobre o que já sabe sobre o tema*. (E7), e [...] *elucidativo, interativo e criativo, motivando o participante ao processo de aprendizagem. Todos os recursos* (vídeos, fluxos e história em quadrinhos) *estão fantásticos!* (E9).

## DISCUSSÃO

A gestão das listas de espera deve ter a participação de vários atores. A integração multiprofissional tem como consequência a melhoria do atendimento ao paciente^(4,18)^. Além disso, realizar o gerenciamento das listas de espera multiprofissional aumenta a garantia de justiça e transparência no processo de atendimento ao paciente eletivo. A existência da lista compõe uma problemática mundial dos serviços públicos de saúde indicado pela OECD^(5-6,8)^ , mas não dos serviços privados, já que arrecadam recursos financeiros a cada internação e/ou procedimento executado.

Juntamente com a falta de orientações legais específicas, existe o problema de escassez de produção literária sobre o tema^(2-3)^. Assim, reflete-se que, apesar de as listas de espera não serem um problema recente de nível nacional e, mesmo, internacional, no Brasil, as tratativas nesse temerário ainda são incipientes. Isso reforça ainda mais a necessidade de ações educativas dos profissionais que atuam no gerenciamento de listas.

Para de fato ter sentido, a educação profissional deve ser capaz de promover transformações efetivas e significativas nas práticas assistenciais e na organização dos processos de trabalho dos serviços de saúde. A educação profissional nas instituições hospitalares deve ser desenvolvida de maneira permanente, ativa e consolidada, contribuindo para a qualificação da assistência para o paciente^(19-20)^. Salienta-se, dessa forma, a necessidade de ações educativas com o objetivo de estimular, acompanhar e fortalecer a qualificação profissional dos trabalhadores que atuam na área da saúde, visando à transformação das práticas de saúde de atendimento com base nos princípios fundamentais do SUS.

A tecnologia educativa foi estruturada para o AVA, que buscou envolver os princípios da aprendizagem colaborativa. Almeja-se que os participantes se envolvam com a sua aprendizagem, a partir das situações reais que foram criadas, e que o curso propicie a articulação do conteúdo com o contexto do trabalho do participante^(21-22)^.

O planejamento foi delineado em dois PAP, que possibilitaram definir os objetivos a serem atingidos pelos participantes do curso, os conteúdos desenvolvidos e os objetos de aprendizagem utilizados em cada módulo (inclusive as estratégias utilizadas para avaliar o progresso dos usuários). Consideram-se como subsídios para definição dos conteúdos nos PAP a literatura científica atualizada e as experiências do público-alvo^(9,11)^.

Destaca-se que, nos cursos autoinstrucionais e *online,* devem ser considerados, ainda no planejamento, os elementos próprios da modalidade a distância, como a seleção das tecnologias e a identidade visual, que serão utilizadas e devem ser contempladas pela descrição em recursos no PAP^(23-24)^. Por isto, já que o tema da gestão das listas de espera é um assunto complexo, foram feitos esforços na elaboração dos objetos de aprendizagem que possibilitem mais dinamicidade e engajamento dos participantes no curso.

Dessa forma, a literatura científica menciona que os *storyboards* são importantes na construção de uma tecnologia educativa, pois eles organizam e estruturam os conteúdos e as atividades em uma sequência lógica na etapa de desenvolvimento^(10)^. Todos os *storyboards* do curso foram avaliados por um comitê de especialistas.

A proposta da tecnologia educativa abrangeu etapas de Análise, Desenho e Desenvolvimento, conforme o modelo ADDIE, que gerou 17 produtos. Esses são considerados como sendo objetos de aprendizagem que sustentam a proposta pedagógica do curso multiprofissional.

Cabe enfatizar que se, no processo de aprendizagem, o conteúdo é construído por meio de palavras e imagens que aguçam a audição e a visão, é possível tornar o aprendizado mais significativo do que se houvesse foco apenas na leitura em tela^(25)^. Recomenda-se que a produção de recursos educativos no formato de vídeo elaborado para compor cursos não exceda dez minutos de duração^(26)^. Em consideração a essa instrução, nenhum dos quatro vídeos e nenhuma das duas HQ animadas criados para o curso excedeu esse tempo de duração. Todos os vídeos encontram-se agrupados em uma *playlist* chamada “Complexo Regulador de Saúde”, disponível no canal do *YouTube* ^®^ do grupo de pesquisa.

O processo de avaliação por um comitê de especialistas fornece suporte de forma criteriosa e cuidadosa às ações educativas planejadas^(10)^. Ressalta-se que os PAP e *storyboards* do curso elaborados atingiram o valor de adequação de conteúdo preconizado pela literatura^(14)^.

Estudo que avaliou recursos educativos digitais para a saúde e segurança no trabalho na Atenção Primária à Saúde e que também utilizou o IVCES encontrou valores entre 0,88 e 0,96, o que demonstra a qualidade do curso^(27)^. A avaliação do desenvolvimento de um curso direcionado ao uso de diagnósticos e intervenções em saúde mental nas unidades de internação clínica-cirúrgica obteve valores de 0,84 a 1,00^(28)^. Outro estudo complementou que especialistas que avaliaram um curso *online* sobre dor neonatal ressaltaram a necessidade de construir conteúdos e disponibilizar produtos educacionais de maneira independente, permitindo ao usuário decidir o itinerário do seu estudo^(29)^.

Entretanto, os itens que constam nos domínios do IVCES são importantes à medida que disponibilizam embasamento de diversos aspectos pertinentes aos conteúdos educativos, assegurando o oferecimento de um produto adequado ao público-alvo^(15)^. Assim, as sugestões, as críticas e os elogios dos especialistas contribuíram para a qualidade do curso proposto.

Todos os objetos de aprendizagem produzidos por este estudo podem ser (re)utilizados de acordo com a necessidade instalada, além de o profissional possuir a autonomia sobre a sequência dos conteúdos que deseja aprender no curso. Ademais, o uso de tecnologias na educação gera um ganho cognitivo superior para os participantes em relação ao ensino convencional^(30-31)^.

O estudo demonstrou relevância ao ser ofertado aos profissionais que atuam no gerenciamento das listas de espera, possibilitando que compreendam os conceitos, reflitam sobre os impactos e aprimorem os conhecimentos operacionais relacionados à gestão em lista de espera de pacientes eletivos cirúrgicos e clínicos.

### Limitações do estudo

Como desafio vivenciado neste estudo, pode-se mencionar a etapa de concepção do modelo ADDIE, em que foram necessárias muitas horas de trabalho para a entrega do produto apresentado. Isso exigiu uma organização da equipe executora que trabalhou de maneira remota, visto a abrangência da pandemia e do atendimento às normas de distanciamento seguro no período da construção da tecnologia educativa.

A exiguidade do tempo para a completude de todas as fases do modelo ADDIE, no qual se inclui a fase de execução não concluída, foi outro fator limitador. Outros dois fatores contribuíram para intensificar o nível de dificuldade da proposta do curso: o fato de a problemática do tema tratado ainda apresentar uma literatura científica incipiente; e a prática do gerenciamento das listas não possuir um processo organizacional comum em todos os serviços de saúde.

### Contribuições para as áreas da enfermagem e saúde

Com base nos fatores limitadores e dificultadores supracitados, acentua-se a importância da oferta do curso para os profissionais de saúde, visto que o uso dessa tecnologia educacional no AVA torna possível esclarecer, atualizar e inspirar novas práticas no gerenciamento de listas de espera. Assim, essa tecnologia educativa contribui para a prática das diferentes categorias profissionais, especialmente as enfermeiras reguladoras, qualificando as competências no gerenciamento de abrangência nacional das listas de espera de pacientes eletivos.

As práticas de regulação e gerenciamento das listas de espera para a enfermagem com atuação em NIR são recentes. No entanto, é necessário discutir sobre esse tema ainda no período de formação acadêmica, quando se trata da gestão em enfermagem e na saúde. Compreende-se que o enfermeiro possui a competência gerencial e de liderança para a mediação do processo de regulação das listas, já que fortalece a análise de indicadores e a formulação de estratégias na instituição^(4)^. Ademais, ressalta-se que as tecnologias educativas possibilitam a qualificação do enfermeiro e de outros profissionais, de modo que esses profissionais possam participar e contribuir em setores em que o trabalho é multidisciplinar.

## CONCLUSÕES

A construção e a avaliação da tecnologia educativa para a formação multiprofissional atingiram a qualidade do conteúdo apresentado, sendo utilizadas diversas estratégias para elaboração de objetos de aprendizagem, que se interrelacionaram na composição pedagógica do curso. A oferta do curso na sua modalidade autoinstrucional favorece a ampliação de saberes à medida que possibilita ao profissional que atua no gerenciamento de listas de espera a autonomia no processo de ensino-aprendizagem. Ademais, contribui para o desenvolvimento e aprimoramento do processo de trabalho, para que a regulação de leitos seja feita de maneira mais qualificada e eficiente.

A participação dos especialistas na avaliação dos módulos do curso foi fundamental para qualificar a tecnologia educativa, bem como avalizou cientificamente, por meio de uma escala, a adequação do conteúdo associado aos objetos de aprendizagem. Entende-se que, para ocorrer aprimoramento e atualização, deve-se incentivar e dispor de tecnologias educativas baseadas em rigor técnico-científico.

Considera-se que essa tecnologia educativa deve destinar-se a estudantes, residentes e profissionais, em especial aos que atuam em instituições de saúde pública. Tais instituições podem estar comumente marcadas pela disparidade da gestão e de condições entre as regiões do Brasil. Dessa forma, o curso de formação multiprofissional como tecnologia educativa pode colaborar com os ODS para reduzir as desigualdades, amenizar as diferenças, além de promover equidade no atendimento e boa saúde ao paciente.
